# The effect of human papillomavirus status on prognosis and local treatment strategies of T1-2N0 oropharyngeal squamous cell cancer

**DOI:** 10.3389/fpubh.2022.900294

**Published:** 2022-07-25

**Authors:** Ping Zhou, Deng-Lin Chen, Chen-Lu Lian, San-Gang Wu, Shi-Yang Zhang

**Affiliations:** ^1^Department of Radiation Oncology, Xiamen Cancer Center, Xiamen Key Laboratory of Radiation Oncology, The First Affiliated Hospital of Xiamen University, School of Medicine, Xiamen University, Xiamen, China; ^2^Department of Medical Oncology, Hainan General Hospital (Hainan Affiliated Hospital of Hainan Medical University), Haikou, China; ^3^Department of Hospital Infection Management, The First Affiliated Hospital of Xiamen University, School of Medicine, Xiamen University, Xiamen, China

**Keywords:** oropharyngeal squamous cell cancer, human papillomavirus, prognosis, radiotherapy, surgery

## Abstract

**Purpose:**

To explore the effect of human papillomavirus (HPV) status on prognosis and further investigate whether human papillomavirus (HPV) status has an impact on the local treatment strategies for T1-2N0 oropharyngeal squamous cell cancer (OPSCC) patients.

**Methods:**

Patients diagnosed with T1-2N0 OPSCC between 2010 and 2015 were included from the Surveillance, Epidemiology, and End Results database. Data were analyzed using propensity score matching (PSM), Chi-square test, Kaplan-Meier survival analysis, and Cox multivariable analyses.

**Results:**

A total of 1,004 patients were identified, of whom 595 (59.3%) had HPV-related tumors. Of all the patients, 386 (38.4%) and 618 (61.6%) received definitive radiotherapy and radical surgery, respectively. HPV status had no significant effect on local treatment strategies for early-stage OPSCC (*P* = 0.817). The 3-year cancer-specific survival (CSS) and overall survival (OS) were 89.6 and 80.1%, respectively. Compared to those with HPV-negative diseases, patients with HPV-positive diseases had better CSS and OS. A total of 222 pairs of patients were completely matched after PSM. The results of multivariate Cox regression analysis showed that patients with HPV-positive disease had significantly better CSS (*P* = 0.001) and OS (*P* < 0.001) compared to those with HPV-negative tumors. However, local treatment strategy was not associated with survival outcomes after PSM (CSS, *P* = 0.771; OS, *P* = 0.440). The subgroup analysis showed comparable CSS and OS between those treated with radical surgery and definitive radiotherapy regardless of HPV status.

**Conclusions:**

HPV status is an independent prognostic factor for the survival of stage T1-2N0 OPSCC patients. Local treatment strategies had no significant effect on the survival of early-stage OPSCC regardless of HPV status. Patients with early-stage OPSCC should be informed regarding the pros and cons of definitive radiotherapy or radical surgery.

## Introduction

The steady rise in the incidence of oropharyngeal cancer (OPC) has aroused people's concern (1). Approximately 93,000 cases were newly diagnosed with OPC and 51,000 cases died worldwide in 2018 (2). Squamous cell carcinoma (SCC) is the most common histological subtype in patients with tumors located in the oropharynx. Human papillomavirus (HPV) may play a leading role in the elevated incidence of OPC (1). The statistics from the United States (US) showed that the proportion of HPV-positive OPC patients increased from 16.3% in the year 1984–1989 to 71.7% in the year 2000–2004 (3). HPV-positive oropharyngeal squamous cell carcinoma (OPSCC) has specific clinical features, including poorly differentiation, lower tumor stage, and higher nodal stage. In addition, HPV-related tumors have better sensitivity to radiotherapy and chemotherapy contributing to a better survival outcome (4–7).

Unlike oral cancer, most patients with OPSCC are diagnosed with locally advanced stage (60 vs. 89%) (8, 9). Although the proportion of early-stage (T1-2N0) OPSCC is relatively low, they have excellent outcomes, especially for those with HPV-positive tumors. In the current National Comprehensive Cancer Network (NCCN) treatment guidelines, radical surgery or definitive radiotherapy is the optional treatment for early-stage OPSCC regardless of the HPV status (10). However, the role of HPV status in local treatment strategies of early-stage OPSCC remains unclear. Since different local treatment strategies have their pros and cons, exploring the effect of different local treatment strategies on survival outcomes according to different HPV status have important clinical implications for early-stage OPSCC. In light of this, our study aimed to explore the effect of HPV status on prognosis and further investigate whether HPV status has an impact on the local treatment strategies for T1-2N0 OPSCC patients.

## Materials and methods

### Database and patient selection criteria

The database of head and neck cancer (HNC) with HPV status was released by the Surveillance, Epidemiology, and End Results (SEER) program in 2018 (11). It includes 40,866 HNC patients including HPV status and treatment strategies from 2010 to 2015. The HPV status in the database were classified as HPV-positive, HPV-negative, and unknown. Patients who met the following criteria were included: (1) stage T1-2N0 OPSCC; (2) diagnosed between 2010 and 2015; (3) known HPV status; (4) received definitive radiotherapy or radical surgery. Patients who were treated with partial excision of the primary site, local tumor excision, or local tumor destruction were excluded. HPV status was tested using immunohistochemistry for p16 and/or polymerase chain reaction (PCR) or *in situ* hybridization (ISH)-based methods of pathologic specimens from either the primary oropharyngeal tumors or the corresponding cervical lymph node metastases. This HPV data set is reviewed by a SEER data-quality team to ensure accuracy (12). This study used a public de-identified SEER database and the institutional review board approval was waived.

### Data collection

Details were selected including age at diagnosis, gender, race, primary tumor sites, tumor grade, tumor (T) stage, HPV status, and local treatment strategies. We included oropharynx cancer involving soft palate, tongue base, pharyngeal tonsil, and oropharynx not otherwise specified (NOS). The seventh edition of the American Joint Committee on Cancer staging system was used to classify TNM staging. Cancer-specific survival (CSS) and overall survival (OS) were selected as the primary endpoints in this study. CSS was defined as the time from the initial diagnosis of OPSCC to the death of head and neck cancer. OS was defined as the time from the initial diagnosis of OPSCC to death from various causes.

### Statistical analysis

The association of categorical variables was compared using the Chi-square test and Fisher's exact test. The Kaplan-Meier methods were used to sketch survival curves and a log-rank test was used to compare the difference in survival outcomes. Multivariable Cox regression models were performed to determine the independent factors associated with CSS and OS. Propensity score matching (PSM) was used to reduce selection bias, including the following characteristics: race, age at diagnosis, gender, primary tumor site, tumor grade, T stage, and HPV status (13). SPSS statistical software (version 25.0, IBM Corporation, Armonk, NY, USA) was used for data analysis. *P* < 0.05 was considered to be statistically significant.

## Results

### Patients' clinicopathological characteristics before PSM

A total of 1,004 patients were included in this study (Table 1). Of these patients, the majority were male (*n* = 753, 75.1%), T2 stage (*n* = 612, 61.0%), and white race (*n* = 905, 90.1%). Tonsil predominated with 53.8% (*n* = 540) of the primary tumor site, followed by tongue base (*n* = 324, 32.3%), palate soft (*n* = 75, 7.5%), and oropharyngx NOS (*n* = 65, 6.5%). Of the 867 patients with available tumor grade, 7.8% (*n* = 68), 44.5% (*n* = 386), and 47.6% (*n* = 413) of patients had well differentiated, moderately differentiated, and poorly or undifferentiated diseases.

**Table 1:** Patients and treatment characteristics by treatment strategies before and after propensity matching analysis.

**Variables**	**Before PSM**				**After PSM**			
	***n* (%)**	**Radiotherapy (%)**	**Surgery (%)**	***P***	***n***	**Radiotherapy**	**Surgery**	***P***
**Race**								
White	905 (90.1)	353 (91.2)	553 (89.5)	0.014	424	212	212	1.000
Black	66 (6.6)	29 (7.5)	37 (6.0)		18	9	9	
Other	33 (3.3)	5 (1.3)	28 (4.5)		2	1	1	
**Age (years)**								
<50	586 (58.4)	211 (54.7)	375 (60.7)	0.060	242	121	121	1.000
≥50	418 (41.6)	175 (45.3)	243 (39.3)		202	101	101	
**Gender**								
Male	753 (75.1)	290 (75.1)	463 (74.9)	0.940	352	176	176	1.000
Female	251 (25.0)	96 (24.9)	155 (25.1)		92	46	46	
**Primary sites**								
Palate soft	75 (7.5)	36 (9.3)	39 (6.3)	<0.001	22	11	11	1.000
Oropharynx NOS	65 (6.5)	37 (9.6)	28 (4.5)		24	12	12	
Tongue base	324 (32.3)	157 (40.4)	167 (27.0)		162	81	81	
Tonsil	540 (53.8)	156 (40.4)	384 (62.1)		236	118	118	
**Differentiation**								
Well differentiated	68 (6.8)	23 (6.0)	45 (7.3)	0.126	14	7	7	1.000
Moderately differentiated	386 (38.4)	146 (37.8)	240 (38.8)		192	96	96	
Poorly/undifferentiated	413 (41.1)	128 (33.2)	285 (46.1)		176	88	88	
Unknown	137 (13.6)	89 (23.1)	48 (7.8)		62	31	31	
**Tumor stage**								
T1	392 (39.0)	102 (26.4)	290 (46.9)	<0.001	132	66	66	1.000
T2	612 (61.0)	284 (73.6)	328 (53.1)		312	156	156	
**HPV status**								
HPV-negative	409 (40.7)	159 (41.2)	250 (40.5)	0.817	186	93	93	1.000
HPV-positive	595 (59.3)	227 (58.8)	368 (59.5)		258	129	129	

Regarding the HPV status, there were 595 (59.3%) patients with HPV-positive tumors and 409 (40.7%) with HPV-negative tumors. The rate of HPV positivity was 52.9, 53.7, 55.9, 64.4, 57.9, and 62.9% from 2010 to 2015, respectively (*P* = 0.210). Patients with HPV-positive disease were more likely to be white race (*P* = 0.001), aged <50 years (*P* < 0.001), male (*P* < 0.001), tumor located in the tonsil (*P* < 0.001), poorly or undifferentiated disease (*P* < 0.001), and T2 stage (*P* = 0.001; Table 2).

**Table 2:** Patient and treatment characteristics by HPV status.

**Variables**	***n***	**HPV-negative (%)**	**HPV-positive (%)**	***P***
**Race**				
White	905	352 (86.1)	553 (92.9)	0.001
Black	66	39 (9.5)	27 (4.5)	
Other	33	18 (4.4)	15 (2.5)	
**Age (years old)**				
<50	586	211 (51.6)	375 (63.0)	<0.001
≥50	418	198 (48.4)	220 (37.0)	
**Gender**				
Male	753	272 (66.5)	481 (80.8)	<0.001
Female	251	137 (33.5)	114 (19.2)	
**Primary sites**				
Palate soft	75	60 (14.7)	15 (2.5)	<0.001
Oropharynx NOS	65	37 (9.0)	28 (4.7)	
Tongue base	324	157 (38.4)	167 (28.1)	
Tonsil	540	155 (37.9)	385 (64.7)	
**Differentiation**				
Well differentiated	68	42 (10.3)	26 (4.4)	<0.001
Moderately differentiated	386	213 (52.1)	173 (29.1)	
Poorly/undifferentiated	413	108 (26.4)	305 (51.3)	
Unknown	137	46 (11.2)	91 (15.3)	
**Tumor stage**				
T1	392	186 (45.5)	206 (34.6)	0.001
T2	612	223 (54.5)	389 (65.4)	
**Treatment strategy**				
Radiotherapy	386	159 (38.9)	227 (38.2)	0.817
Surgery	618	250 (61.1)	368 (61.8)	

### Local treatment strategies

Of all the patients, 618 (61.6%) received radical surgery, and 386 (38.4%) were treated with definitive radiotherapy. In patients who received radical surgery, 274 (44.3%) received additional post-operative radiotherapy, and one (0.2%) patient received intraoperative irradiation. Moreover, in patients who received definitive radiotherapy (*n* = 386), two patients were treated by a combination of the beam with implants or isotopes. Patients with white race (*P* = 0.014), tumor located in the tongue base (*P* < 0.001), and T2 stage (*P* < 0.001) were more likely to receive definitive radiotherapy. Patients with T2 disease had a higher rate of post-operative radiotherapy than those with T1 disease (*P* < 0.001). However, HPV status had no significant effect on local treatment strategies for early-stage OPSCC (*P* = 0.817).

**Figure 1:**
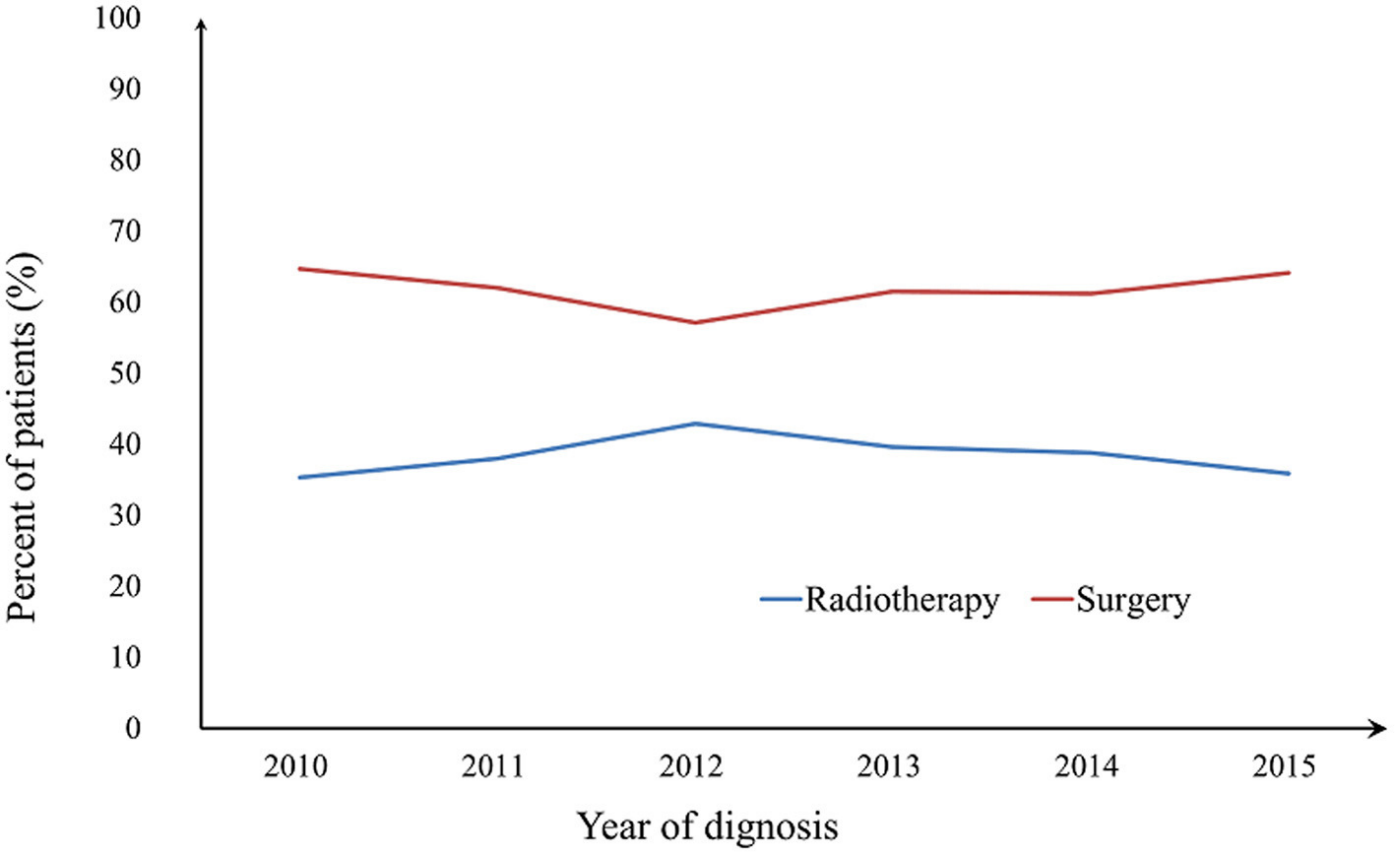
Patients' treatment strategies according to the year of diagnosis.

The proportion of local treatment strategies over time has been depicted in Figure 1. There was no significant difference in the receipt of definitive radiotherapy or radical surgery over time (*P* = 0.794), and there was also no significant difference between the two treatment arms over time regardless of HPV status (HPV-negative, *P* = 0.525; HPV-positive, *P* = 0.637). Similar results were found after stratification by T stage (all *P* > 0.05).

### Survival

**Figure 2:**
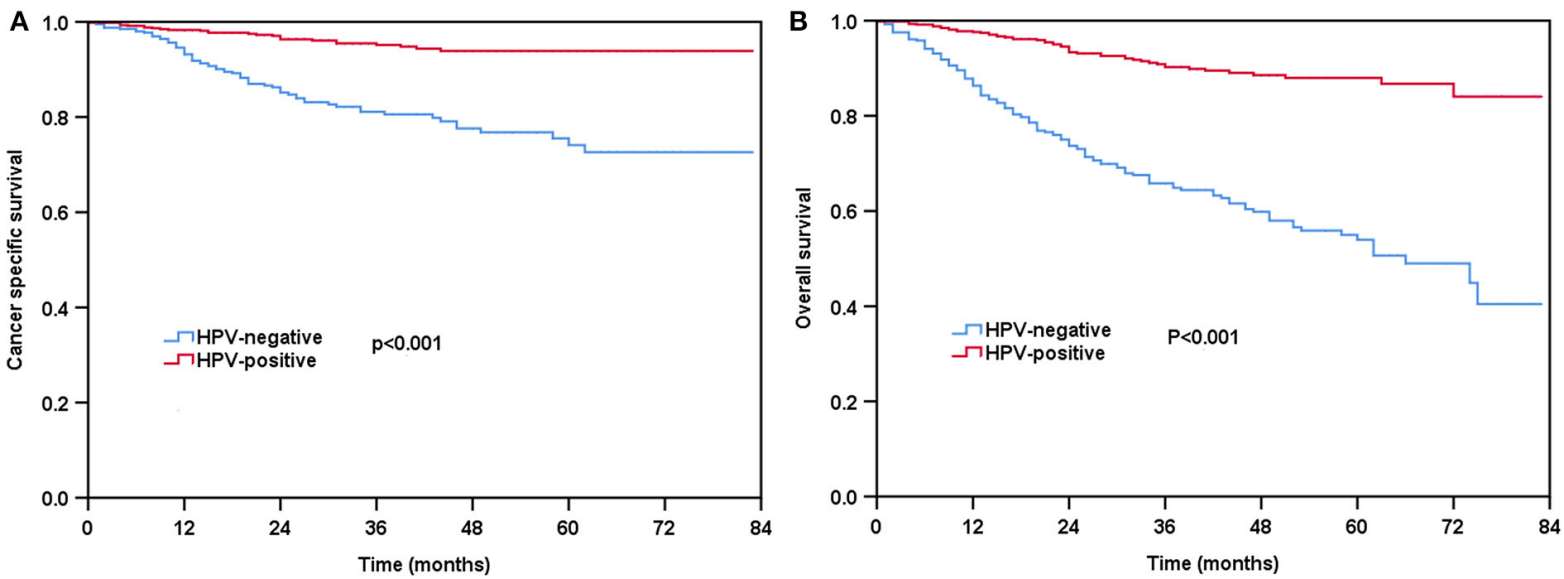
Kaplan-Meier plots of cancer-specific survival **(A)** and overall survival **(B)** by HPV status before propensity score matching (PSM).

With a median follow-up of 31 months (range, 0–83 months), 96 patients died from head and neck cancer, including 70 patients with HPV-negative tumors. The 3-year CSS and OS were 89.6 and 80.1%, respectively. Compared to those with HPV-negative diseases, patients with HPV-positive diseases had better survival outcomes. The 3-year CSS was 95.2 and 81.1% in those with HPV-positive and HPV-negative diseases, respectively (*P* < 0.001, Figure 2A). The 3-year OS was 90.9 and 65.7% in those with HPV-positive and HPV-negative diseases, respectively (*P* < 0.001, Figure 2B).

### Prognostic analyses before and after PSM

Multivariate Cox regression analyses were used to determine prognostic factors related to CSS and OS (Table 3). The results showed that black race [hazard ratio (HR) 2.130, 95% confidence interval (CI) 1.185–3.831, *P* = 0.012], age ≥50 years (HR 1.569, 95%CI 1.043–2.361, *P* = 0.031), T2 stage (HR 1.653, 95%CI 1.050–2.601, *P* = 0.030) and HPV-negative status (HR 3.795, 95%CI 2.317–6.215, *P* < 0.001) were the independent prognostic factors associated with inferior CSS. Regarding OS, the results indicated that black race (HR 1.734, 95%CI 1.110–2.708, *P* = 0.016), age ≥50 years (HR 2.014, 95%CI 1.509–2.687, *P* < 0.001), and HPV-negative status (HR 3.782, 95%CI 2.676–5.343, *P* < 0.001) were the independent prognostic factors associated with inferior OS. Patients with primary tumors located in the tonsil (HR 0.597, 95%CI 0.380–0.939, *P* = 0.026) had better OS compared to those with tumors located in the palate soft. However, the local treatment strategy was not associated with survival outcomes in the multivariate analysis before PSM.

**Table 3:** Multivariate Cox analysis for overall survival and cancers specific survival before and after propensity matching analysis.

**Variables**	**Before PSM**				**After PSM**			
	**OS**		**CSS**		**OS**		**CSS**	
	**HR (95% CI)**	***P***	**HR (95% CI)**	***P***	**HR (95% CI)**	***P***	**HR (95% CI)**	***P***
**Race**								
White	1		1		1		1	
Black	1.734 (1.110–2.708)	0.016	2.130 (1.185–3.831)	0.012	1.027 (0.363–2.906)	0.959	1.760 (0.506–6.120)	0.374
Other	0.724 (0.295–+1.778)	0.481	0.580 (0.141–2.381)	0.449	—	0.970	—	0.981
**Age (years old)**								
<50	1		1		1		1	
≥50	2.014 (1.509–2.687)	0.000	1.569 (1.043–2.361)	0.031	1.843 (1.165–2.915)	0.009	1.293 (0.653–2.561)	0.461
**Gender**								
Male	1				1		1	
Female	1.160 (0.858–1.567)	0.335	1.410 (0.923–2.154)	0.112	1.190 (0.722–1.963)	0.495	1.715 (0.835–3.524)	0.142
**Primary sites**								
Palate soft	1		1		1		1	
Oropharynx NOS	0.549 (0.283–1.067)	0.077	0.557 (0.188–1.650)	0.291	0.454 (0.124–1.661)	0.232	1.082 (0.129–9.088)	0.942
Tongue base	0.802 (0.515–1.249)	0.328	1.211 (0.617–2.379)	0.578	0.827 (0.360–1.900)	0.655	1.548 (0.332–7.211)	0.578
Tonsil	0.597 (0.380–0.939)	0.026	0.685 (0.337–1.391)	0.295	0.650 (0.280–1.511)	0.317	0.969 (0.200–4.708)	0.969
**Differentiation**								
Well differentiated	1		1		1		1	
Moderately differentiated	1.185 (0.703–1.997)	0.524	1.324 (0.593–2.957)	0.494	0.456 (0.172–1.210)	0.115	0.457 (0.099–2.097)	0.338
Poorly/undifferentiated	1.058 (0.607–1.844)	0.824	1.343 (0.584–3.086)	0.488	0.473 (0.166–1.353)	0.163	0.451 (0.088–2.301)	0.365
**Tumor stage**								
T1	1		1		1		1	
T2	1.079 (0.796–1.463)	0.625	1.653 (1.050–2.601)	0.030	1.103 (0.665–1.831)	0.703	2.667 (1.088–6.537)	0.032
**Treatment strategy**								
Radiotherapy	1		1		1		1	
Surgery	0.768 (0.570–1.035)	0.083	0.983 (0.641–1.507)	0.936	0.840 (0.541–1.306)	0.44	1.102 (0.572–2.125)	0.771
**HPV status**								
HPV-positive	1		1		1		1	
HPV-negative	3.782 (2.676–5.343)	0.000	3.795 (2.317–6.215)	0.000	4.188 (2.396–7.322)	0.000	4.399 (1.889–10.245)	0.001

A total of 222 pairs of patients were completely matched after PSM (Table 1). The results of multivariate Cox regression analysis showed that patients with HPV-positive disease had significantly better CSS (HR 0.227, 95%CI 0.098–0.529, *P* = 0.001) and OS (HR 0.239, 95%CI 0.137–0.417, *P* < 0.001) compared to those with HPV-negative tumors. In addition, T stage and age at diagnosis were also the independent prognostic factors associated with survival outcomes. However, local treatment strategy was also not associated with survival outcomes after PSM.

### Effect of local treatment strategies on survival according to HPV status before and after PSM

**Figure 3:**
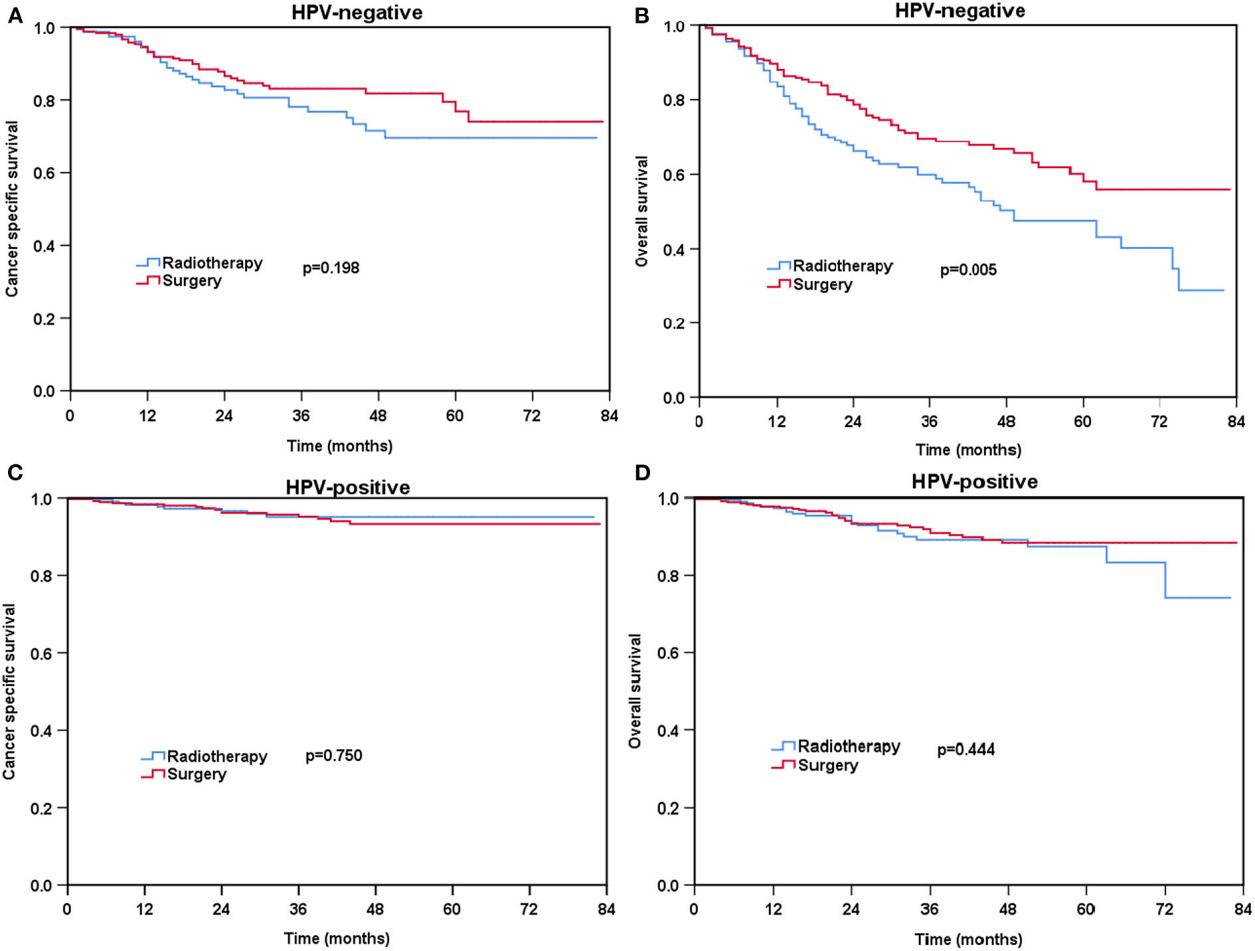
Kaplan-Meier plots of cancer-specific survival **(A)** and overall survival **(B)** for HPV-negative patients and cancer-specific survival **(C)** and overall survival **(D)** for HPV-positive patients before PSM.

**Figure 4:**
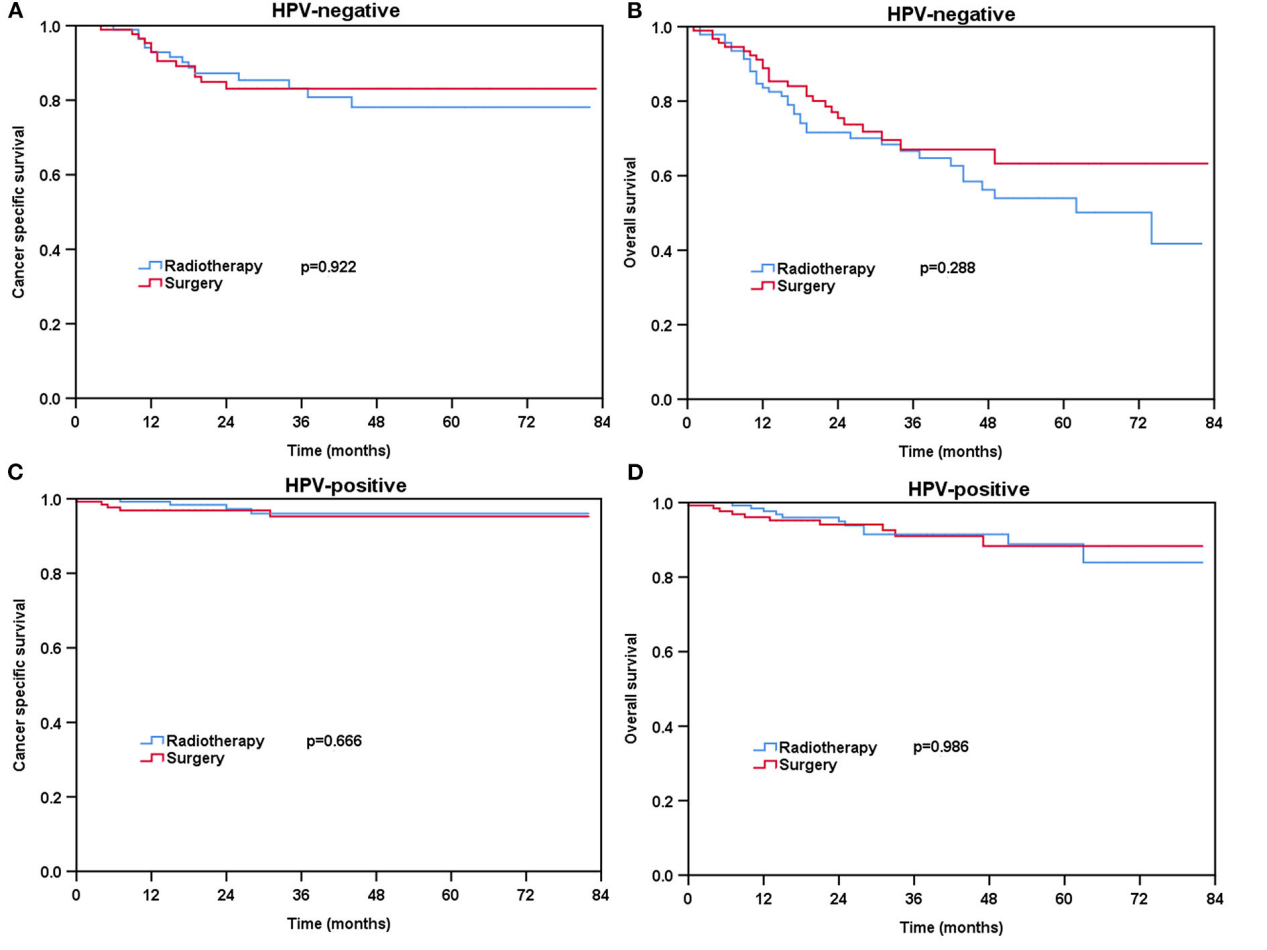
Kaplan-Meier plots of cancer-specific survival **(A)** and overall survival **(B)** for HPV-negative patients and cancer-specific survival **(C)** and overall survival **(D)** for HPV-positive patients after PSM.

For patients with HPV-negative diseases, there was no significant difference in CSS between the radical surgery group and definitive radiotherapy group (*P* = 0.198) (Figure 3A) before PSM. However, patients treated with radical surgery had better OS compared to those treated with definitive radiotherapy (*P* = 0.005) (Figure 3B). A total of 93 pairs of HPV-negative patients were completely matched after PSM. The results also showed comparable CSS (surgery vs. radiotherapy: *P* = 0.922) and OS (surgery vs. radiotherapy: *P* = 0.288) between those treated with radical surgery and definitive radiotherapy after PSM (Figures 4A,B).

Regarding HPV-positive tumors, there was no significant difference in CSS (*P* = 0.750) and OS (*P* = 0.444) for patients treated with definitive radiotherapy compared to those treated with radical surgery before PSM (Figures 3C,D). A total of 129 pairs of HPV-positive patients were completely matched after PSM. Similar results also found after PSM (CSS: *P* = 0.666; OS: *P* = 0.986) (Figures 4C,D).

## Discussion

In our study, we aimed to identify the effect of HPV status on prognosis and local treatment strategies in stage T1-2N0 OPSCC patients. We found that patients with HPV-related OPSCC had better survival outcomes compared to those with HPV-negative tumors. However, local treatment strategies had no significant effect on survival outcomes regardless of HPV status.

The percentage of HPV-related OPSCC was 44.8% of OPSCC patients worldwide according to a large meta-analysis (14). There were significant differences in the rate of HPV positivity between the Eastern and Western countries. The prevalence can reach up to about 60–74.5% in several Western countries including Sweden, Denmark, and the US (15–17). However, in the Eastern countries including China and Japan, only 20–40% of patients were diagnosed with HPV-related OPSCC (18, 19). These findings can be explained by regional differences in HPV infection rates. In our study, using the data from the US SEER program, there were 59.3% of patients suffered from HPV-related OPSCC. We should notice that the proportion of HPV-related OPSCC has increased from 16.3 to 71.7% over the past 15 years in the US (3). Although there were no statistically significant differences, we found that the HPV positivity rate increased from 52.9% in 2010 to 62.9% in 2015.

Similar to the previous studies, we also found that patients with HPV-related OPSCC were more likely to be male, younger, with poorly or undifferentiated disease, and with tumors located in the tonsil (3–5, 20–22). In those with HPV-OPSCC, male patients were more likely than female patients to have an HPV infection (80.8 vs. 19.2%). This may be due to the fact that the HPV viral load of the female genital mucosa is higher HPV than that of the male genital mucosa/skin, and thus men who perform oral sex to women have higher viral dose exposures than vice versa (23, 24). However, patients with HPV-related OPSCC were less likely to have a history of tobacco and alcohol use compared to HPV-negative OPSCC patients (24). Therefore, HPV-positive OPC has specific demographic and clinicopathological features. However, prior studies have shown that patients with HPV-positive tumors were more likely to be smaller primary tumors (25, 26), while more patients with HPV-related tumors had a higher incidence of larger tumor size (T2 stage) than those with HPV-negative tumors in our study. We only included patients with stage T1-2 and node-negative diseases in this study, which was not consistent with the above studies that included patients with stage T1-4N0-3 disease (25, 26). Moreover, it is well known that HPV status is a favorable prognostic factor, HPV-positive OPSCC patients have a better survival outcome compared to HPV-negative patients (7). In this study, we included patients with stage T1-2N0 OPSCC, we also found those with HPV-related tumors had significantly better survival outcomes than those with HPV-negative tumors.

In the current treatment recommendation from the NCCN guidelines, radical surgery or definitive radiotherapy is an optional treatment despite HPV status in early-stage OPSCC (11). Similar treatment strategies are currently recommended in the new version of the Chinese Society of Clinical Oncology (CSCO) guidelines (27). Radiotherapy is a non-invasive technique to cure head and neck cancer patients with organ preservation. However, radiotherapy to the head and neck region may also have several short-term and long-term side effects, including radiation-induced oral mucositis and xerostomia (28, 29). In the contemporary radiotherapy era, those side effects have greatly decreased because the dose to the organs at risk has been significantly reduced (30). The results from Di Gravio et al. showed that patients receiving modern intensity-modulated radiation therapy had outstanding survival and low rates of severe toxicity (31). Meanwhile, minimally transoral robotic surgery (TORS) has been rapidly developed for the treatment of OPSCC which could reduce the toxicity of surgery due to incisions including tracheostomy and deglutition (32–34). According to the study by Cracchiolo et al. using National Cancer Data Base (NCDB), they found that the proportion of surgical treatment increased from 56% in 2004 to 82% in 2013 because the US Food and Drug Administration approved TORS in 2009 (35).

In our cohort, the trend that radical surgery was the first choice for the majority of patients (57.1–64.7%) was stable over time. The ORATOR study compared the quality of life (QOL) and survival outcome between TORS and radiotherapy (RT) for T1-T2N0-2 OPSCC patients (88% of patients had HPV-related tumors) (36). Longitudinal QOL scores were statistically superior after radiotherapy. However, TORS and radiotherapy had differing toxicity profiles, but comparable long-term survival outcomes. TORS group started to use more nutritional supplements at 3 years, while dry mouth scores were higher in the radiotherapy group over time. We should notice that 71% of patients received additional post-operative radiotherapy after TORS in the ORATOR study. In our cohort, 44.5% of patients received additional radiotherapy after radical surgery. Kelly et al. also found that up to 59.1% of OPSCC patients with positive margins and/or extracapsular extension received additional concurrent chemoradiotherapy after receiving surgery (37). Post-operative radiotherapy can bring additional side effects, and ~50% of early-stage OPSCC still requires post-operative radiotherapy. Therefore, patients with early-stage OPSCC should be informed regarding the pros and cons of both treatment options before treatment begins.

In our study, we also investigated whether the HPV status would impact the local treatment strategies in early-stage OPSCC, and we found similar survival between definitive radiotherapy and radical surgery after PSM regardless of HPV status. Two previous studies included stage T1-2N1-2b OPSCC, both came to the same conclusion that the OS was comparable between surgery and definitive radiotherapy regardless of HPV status (37, 38). Thus, HPV status is more indicative of prognosis rather than being included in treatment strategies for OPSCC. There were amounts of studies aiming at reducing the treatment intensity appropriately for HPV-positive OPSCC to improve QOL, without impairing survival outcomes (39).

There are several limitations to the content of this study. First, although we have used PSM analysis to minimize potential selection bias, our findings were from a retrospective observational study and the treatment was not randomized. Second, it was not unambiguous of HPV testing methods and whether HPV status was identified before or after the surgery. In the SEER database regarding HPV status, p16, PCR, or ISH methods were used to determine the status of HPV infection. However, the specific testing methods for HPV status are not recorded in the SEER database. A previous study showed high concordance among the three diagnostic tests, with sensitivity and specificity of 88–97% and 82–88% for the three HPV testing methods, respectively (40). Furthermore, several factors that may influence the prognosis were not recorded, including the status of resection margin, smoking, and alcohol consumption. Moreover, the radiotherapy technique, as well as the surgical technique, were also not recorded in the SEER database. Finally, the patterns of locoregional and distant recurrence were also not included in the SEER database.

## Conclusions

HPV status is an independent prognostic factor for the survival of stage T1-2N0 OPSCC patients. Local treatment strategies had no significant effect on the survival of early-stage OPSCC regardless of HPV status. Patients with early-stage OPSCC should be informed regarding the pros and cons of definitive radiotherapy or radical surgery.

## Data availability statement

The datasets presented in this article are not readily available because the data we obtain comes from the public SEER database (www.seer.cancer.gov). Requests to access the datasets should be directed to www.seer.cancer.gov.

## Ethics statement

Ethical review and approval was not required for the study on human participants in accordance with the local legislation and institutional requirements. Written informed consent for participation was not required for this study in accordance with the national legislation and the institutional requirements.

## Author contributions

PZ, D-LC, and C-LL drafted the manuscript. S-GW acquired the datasets and conducted the statistical analyses. S-GW and S-YZ conceived of the study and participated in the study design. All authors read and approved the final manuscript.

## Funding

This work was partly supported by the Commission Young and Middle-Aged Talents Training Project of Fujian Health Commission (No. 2019-ZQNB-25), National Natural Science Foundation of China (No. 81772893), Natural Science Foundation of Fujian Province (No. 2020J011220), and the Key Medical and Health Projects in Xiamen (No. 3502Z20209002).

## Conflict of interest

The authors declare that the research was conducted in the absence of any commercial or financial relationships that could be construed as a potential conflict of interest.

## Publisher's Note

All claims expressed in this article are solely those of the authors and do not necessarily represent those of their affiliated organizations, or those of the publisher, the editors and the reviewers. Any product that may be evaluated in this article, or claim that may be made by its manufacturer, is not guaranteed or endorsed by the publisher.

## References

[1] Chaturvedi AK Anderson WF Lortet-Tieulent J Curado MP Ferlay J Franceschi S. Worldwide trends in incidence rates for oral cavity and oropharyngeal cancers. J Clin Oncol. (2013) 31:4550–9. 10.1200/JCO.2013.50.3870 24248688 PMC3865341

[2] Bray F Ferlay J Soerjomataram I Siegel RL Torre LA Jemal A. Global cancer statistics 2018: GLOBOCAN estimates of incidence and mortality worldwide for 36 cancers in 185 countries. CA Cancer J Clin. (2018) 68:394–424. 10.3322/caac.21492 30207593

[3] Chaturvedi AK Engels EA Pfeiffer RM Hernandez BY Xiao W Kim E. Human papillomavirus and rising oropharyngeal cancer incidence in the United States. J Clin Oncol. (2011) 29:4294–301. 10.1200/JCO.2011.36.4596 21969503 PMC3221528

[4] Klussmann JP Weissenborn SJ Wieland U Dries V Eckel HE Pfister HJ. Human papillomavirus-positive tonsillar carcinomas: a different tumor entity? Med Microbiol Immunol. (2003) 192:129–32. 10.1007/s00430-002-0126-1 12920586

[5] Huang SH Perez-Ordonez B Liu FF Waldron J Ringash J Irish J. Atypical clinical behavior of p16-confirmed HPV-related oropharyngeal squamous cell carcinoma treated with radical radiotherapy. Int J Radiat Oncol Biol Phys. (2012) 82:276–83. 10.1016/j.ijrobp.2010.08.031 20950953

[6] Klussmann JP Mooren JJ Lehnen M Claessen SM Stenner M Huebbers CU. Genetic signatures of HPV-related and unrelated oropharyngeal carcinoma and their prognostic implications. Clin Cancer Res. (2009) 15:1779–86. 10.1158/1078-0432.CCR-08-1463 19223504

[7] Blitzer GC Smith MA Harris SL Kimple RJ. Review of the clinical and biologic aspects of human papillomavirus-positive squamous cell carcinomas of the head and neck. Int J Radiat Oncol Biol Phys. (2014) 88:761–70. 10.1016/j.ijrobp.2013.08.029 24606845 PMC3990872

[8] Quer M León X Orús C Recher K Gras JR. Análisis de 2.500 carcinomas escamosos de cabeza y cuello [Analysis of 2,500 squamous cell carcinoma of the head and neck]. Acta Otorrinolaringol Esp. (2001) 52:201–5. 10.1016/S0001-6519(01)78198-8 11526864

[9] Rotsides JM Oliver JR Moses LE Tam M Li Z Schreiber D. Socioeconomic and racial disparities and survival of human papillomavirus-associated oropharyngeal squamous cell carcinoma. Otolaryngol Head Neck Surg. (2021) 164:131–8. 10.1177/0194599820935853 32660368

[10] National Comprehensive Cancer Network. NCCN Clinical Practice Guidelines in Oncology: Head and Neck Cancer. V. 1. 2021. Available online at: https://www.nccn.org/professionals/physician_gls/pdf/head_and_neck.pdf (accessed January 15, 2021).

[11] Surveillance, Epidemiology, and End Results (SEER) Program. SEER*Stat Database: Incidence - SEER 18 Regs Custom Data Head and Neck (Select Schemas with HPV Recode and Additional Treatment Fields), Nov 2018 Sub (2010-2016) - Linked To County Attributes - Total U.S., 1969-2017 Counties, National Cancer Institute, DCCPS, Surveillance Research Program, released April 2019, based on the November 2018 submission. Available online at: www.seer.cancer.gov

[12] Fullerton ZH Butler SS Mahal BA Muralidhar V Schoenfeld JD Tishler RB. Short-term mortality risks among patients with oropharynx cancer by human papillomavirus status. Cancer. (2020) 126:1424–33. 10.1002/cncr.32652 31930488

[13] Rosenbaum RR Rubin DB. Constructing a control group using multivariate matched sampling methods that incorporate the propensity score. Am Stat. (1985) 39:33–8. 10.1080/00031305.1985.10479383

[14] Mariz BALA Kowalski LP William WN Jr. de Castro G Jr. Chaves ALF Santos M. Global prevalence of human papillomavirus-driven oropharyngeal squamous cell carcinoma following the ASCO guidelines: a systematic review and meta-analysis. Crit Rev Oncol Hematol. (2020) 156:103116. 10.1016/j.critrevonc.2020.103116 33115701

[15] Haeggblom L Attoff T Yu J Holzhauser S Vlastos A Mirzae L. Changes in incidence and prevalence of human papillomavirus in tonsillar and base of tongue cancer during 2000-2016 in the Stockholm region and Sweden. Head Neck. (2019) 41:1583–90. 10.1002/hed.25585 30584688

[16] Carlander AF Grønhøj Larsen C Jensen DH Garnæs E Kiss K Andersen L. Continuing rise in oropharyngeal cancer in a high HPV prevalence area: a Danish population-based study from 2011 to 2014. Eur J Cancer. (2017) 70:75–82. 10.1016/j.ejca.2016.10.015 27888679

[17] D'Souza G Westra WH Wang SJ van Zante A Wentz A Kluz N. Differences in the prevalence of human papillomavirus (HPV) in head and neck squamous cell cancers by sex, race, anatomic tumor site, and HPV detection method. JAMA Oncol. (2017) 3:169–77. 10.1001/jamaoncol.2016.3067 27930766 PMC7286346

[18] Lam EW Chan JY Chan AB Ng CS Lo ST Lam VS. Prevalence, clinicopathological characteristics, and outcome of human papillomavirus-associated oropharyngeal cancer in southern chinese patients. Cancer Epidemiol Biomarkers Prev. (2016) 25:165–73. 10.1158/1055-9965.EPI-15-0869 26604268

[19] Yamashita Y Ikegami T Hirakawa H Uehara T Deng Z Agena S. Staging and prognosis of oropharyngeal carcinoma according to the 8th Edition of the American Joint Committee on Cancer Staging Manual in human papillomavirus infection. Eur Arch Otorhinolaryngol. (2019) 276:827–36. 10.1007/s00405-018-05263-x 30594962 PMC6411679

[20] Windon MJ D'Souza G Rettig EM Westra WH van Zante A Wang SJ. Increasing prevalence of human papillomavirus-positive oropharyngeal cancers among older adults. Cancer. (2018) 124:2993–9. 10.1002/cncr.31385 29710393 PMC6033632

[21] Gillison ML Koch WM Capone RB Spafford M Westra WH Wu L. Evidence for a causal association between human papillomavirus and a subset of head and neck cancers. J Natl Cancer Inst. (2000) 92:709–20. 10.1093/jnci/92.9.709 10793107

[22] Taberna M Mena M Pavón MA Alemany L Gillison ML Mesía R. Human papillomavirus-related oropharyngeal cancer. Ann Oncol. (2017) 28:2386–98. 10.1093/annonc/mdx304 28633362

[23] Gillison ML Broutian T Pickard RK Tong ZY Xiao W Kahle L. Prevalence of oral HPV infection in the United States, 2009-2010. JAMA. (2012) 307:693–703. 10.1001/jama.2012.101 22282321 PMC5790188

[24] Pytynia KB Dahlstrom KR Sturgis EM. Epidemiology of HPV-associated oropharyngeal cancer. Oral Oncol. (2014) 50:380–6. 10.1016/j.oraloncology.2013.12.019 24461628 PMC4444216

[25] Ang KK Harris J Wheeler R Weber R Rosenthal DI Nguyen-Tân PF. Human papillomavirus and survival of patients with oropharyngeal cancer. N Engl J Med. (2010) 363:24–35. 10.1056/NEJMoa0912217 20530316 PMC2943767

[26] Lewis JS Jr. Morphologic diversity in human papillomavirus-related oropharyngeal squamous cell carcinoma: Catch Me If You Can! Mod Pathol. (2017) 30:S44–53. 10.1038/modpathol.2016.152 28060372

[27] CSCO Guidelines for Head and Neck Cancer. Chinese Society of Clinical Oncology (CSCO) diagnosis and treatment guidelines for head and neck cancer 2018 (English version). Chin J Cancer Res. (2019) 31:84–98. 10.21147/j.issn.1000-9604.2019.01.05 30996568 PMC6433588

[28] Shih A Miaskowski C Dodd MJ Stotts NA MacPhail L. Mechanisms for radiation-induced oral mucositis and the consequences. Cancer Nurs. (2003) 26:222–9. 10.1097/00002820-200306000-00008 12832955

[29] Vissink A Jansma J Spijkervet FK Burlage FR Coppes RP. Oral sequelae of head and neck radiotherapy. Crit Rev Oral Biol Med. (2003) 14:199–212. 10.1177/154411130301400305 12799323

[30] Chao KS Majhail N Huang CJ Simpson JR Perez CA Haughey B. Intensity-modulated radiation therapy reduces late salivary toxicity without compromising tumor control in patients with oropharyngeal carcinoma: a comparison with conventional techniques. Radiother Oncol. (2001) 61:275–80. 10.1016/S0167-8140(01)00449-2 11730997

[31] Di Gravio EJ Lang P Kim HAJ Chinnery T Mundi N MacNeil SD. Modern treatment outcomes for early T-stage oropharyngeal cancer treated with intensity-modulated radiation therapy at a tertiary care institution. Radiat Oncol. (2020) 15:261. 10.1186/s13014-020-01705-1 33168055 PMC7654053

[32] Chen MM Roman SA Kraus DH Sosa JA Judson BL. Transoral robotic surgery: a population-level analysis. Otolaryngol Head Neck Surg. (2014) 150:968–75. 10.1177/0194599814525747 24618503

[33] Moore EJ Olsen SM Laborde RR García JJ Walsh FJ Price DL. Long-term functional and oncologic results of transoral robotic surgery for oropharyngeal squamous cell carcinoma. Mayo Clin Proc. (2012) 87:219–25. 10.1016/j.mayocp.2011.10.007 22386176 PMC3538408

[34] Park YM Kim WS Byeon HK Lee SY Kim SH. Oncological and functional outcomes of transoral robotic surgery for oropharyngeal cancer. Br J Oral Maxillofac Surg. (2013) 51:408–12. 10.1016/j.bjoms.2012.08.015 23063012

[35] Cracchiolo JR Baxi SS Morris LG Ganly I Patel SG Cohen MA. Increase in primary surgical treatment of T1 and T2 oropharyngeal squamous cell carcinoma and rates of adverse pathologic features: national cancer data base. Cancer. (2016) 122:1523–32. 10.1002/cncr.29938 26970050 PMC4860079

[36] Nichols AC Theurer J Prisman E Read N Berthelet E Tran E. randomized trial of radiotherapy versus transoral robotic surgery for oropharyngeal squamous cell carcinoma: long-term results of the ORATOR trial. J Clin Oncol. (2022) 40:866–75. 10.1200/JCO.21.01961 34995124

[37] Kelly JR Park HS An Y Contessa JN Yarbrough WG Burtness BA. Comparison of survival outcomes among human papillomavirus-negative cT1-2 N1-2b patients with oropharyngeal squamous cell cancer treated with upfront surgery vs. definitive chemoradiation therapy: an observational study. JAMA Oncol. (2017) 3:1107–111. 10.1001/jamaoncol.2016.5769 28056116 PMC5824218

[38] Kelly JR Park HS An Y Yarbrough WG Contessa JN Decker R. Upfront surgery versus definitive chemoradiotherapy in patients with human Papillomavirus-associated oropharyngeal squamous cell cancer. Oral Oncol. (2018) 79:64–70. 10.1016/j.oraloncology.2018.02.017 29598952

[39] Kelly JR Husain ZA Burtness B. Treatment de-intensification strategies for head and neck cancer. Eur J Cancer. (2016) 68:125–33. 10.1016/j.ejca.2016.09.006 27755996 PMC5734050

[40] Schache AG Liloglou T Risk JM Filia A Jones TM Sheard J. Evaluation of human papilloma virus diagnostic testing in oropharyngeal squamous cell carcinoma: sensitivity, specificity, and prognostic discrimination. Clin Cancer Res. (2011) 17:6262–71. 10.1158/1078-0432.CCR-11-0388 21969383 PMC3188400

